# Characterization and Evaluation of Transgenic Rice Pyramided with the *Pi* Genes *Pib*, *Pi25* and *Pi54*

**DOI:** 10.1186/s12284-021-00512-w

**Published:** 2021-09-07

**Authors:** Meifang Peng, Xiaomin Lin, Xiaoli Xiang, Huibo Ren, Xiaoli Fan, Kegui Chen

**Affiliations:** Institute of Biotechnology and Nuclear Technology, Sichuan Academy of Agricultural Sciences, 106 Shizishan Road, Chengdu, 610061 Sichuan China

**Keywords:** *O. sativa*, *Pib*, *Pi25*, *Pi54*, Blast disease, Transgenesis, Gene pyramiding, RNA-Seq, Transcriptome

## Abstract

**Background:**

Emergence of new pathogen strains of *Magnaporthe oryzae* is a major reason for recurrent failure of the resistance mediated by a single resistance gene (*Pi*) in rice. Stacking various *Pi* genes in the genome through marker-assisted selection is thus an effective strategy in rice breeding for achieving durable resistance against the pathogen. However, the effect of pyramiding of multiple *Pi* genes using transgenesis still remains largely unknown.

**Results:**

Three *Pi* genes *Pib*, *Pi25* and *Pi54* were transferred together into two rice varieties, the *indica* variety Kasalath and the *japonica* variety Zhenghan 10. Transgenic plants of both Kasalath and Zhenghan 10 expressing the *Pi* transgenes showed imparted pathogen resistance. All the transgenic lines of both cultivars also exhibited shorter growth periods with flowering 2–4 days early, and shorter plant heights with smaller panicle. Thus, pyramiding of the *Pi* genes resulted in reduced grain yields in both rice cultivars. However, tiller numbers and grain weight were generally similar between the pyramided lines and corresponding parents. A global analysis of gene expression by RNA-Seq suggested that both enhancement and, to a lesser extent, inhibition of gene transcription occurred in the pyramided plants. A total of 264 and 544 differentially expressed genes (DEGs) were identified in Kasalath and Zhenghan 10, respectively. Analysis of the DEGs suggested that presence of the *Pi* transgenes did not alter gene expression only related to disease resistance, but also impacted many gene transcriptions in the pathways for plant growth and development, in which several were common for both Kasalath and Zhenghan 10.

**Conclusion:**

Pyramiding of the *Pi* genes *Pib*, *Pi25* and *Pi54* via transgenesis is a potentially promising approach for improving rice resistance to the pathogen *Magnaporthe oryzae*. However, pleiotropic effects of the *Pi* genes could potentially result in yield loss. These findings support the idea that immunity is often associated with yield penalties. Rational combination of the *Pi* genes based on the genetic background may be important to balance yield and disease resistance.

**Supplementary Information:**

The online version contains supplementary material available at 10.1186/s12284-021-00512-w.

## Background

Hemibiotrophic fungus *Magnaporthe oryzae* is able to infect entire rice (*O. sativa* L.) plant, resulting in blast disease, typically causing up to a 30% loss of the annual harvest. Under severe epiphytic conditions it may even result in grain loss between 70 and 90% in isolated fields (Dean et al. 2012). Rice is a global crop feeding approximately 50% of the population. To meet increasing food demand due to the population growth, concerted efforts are required to increase rice productivity by minimizing the occurrence of disease epidemics and reducing yield losses. Development of the disease resistant cultivars by introduction of resistance (R) genes into elite rice varieties has been demonstrated to be an effective and sustainable approach (Miah et al. 2013).

In rice, the first *R* gene identified was *Pib* in the *japonica* variety in (Wang et al. 1999). So far, approximately 100 resistance *R* genes/alleles designed as *Pi* genes and 500 quantitative trait loci associated with blast resistance have been reported, of which 25 *Pi* genes have been successfully cloned and characterized (Li W et al., 2019). However, most of the *Pi* genes confer resistance to one or a few isolates of *M. oryzae* in the gene-for-gene model. A rice cultivar with such a *Pi* gene tends to lose its resistance in a few years of cultivation because the fungus evolves rapidly (Jia et al. 2000; Valent and Khang, 2010).

The combination of multiple *R* genes could weaken selection pressure on the respective pathogen and is an effective method to breed a cultivar with durable resistance (Joshi and Nayak 2010). Thus, various molecular markers linked to *R* genes have been invented and marker-assisted selection (MAS) transferring a specific *Pi* allele at its target locus from donor into recipient has been successfully used to confer blast resistance to susceptible cultivars (Liu et al. 2002; Narayanan et al. 2002; Wu et al. 2016). Based on the same principle, two or more genes were further pyramided together, such as *Pib* and *Pita* (Fujita et al. 2009) and *Pi1*, *Piz-5* and *Pita* (Hittalmani et al. 2000).

MAS technology combined with backcrossing is known as marker-assisted backcrossing, which is a currently effective approach for obtaining durable resistance against *M. oryzae* (Ashkani et al. 2015). However, it is obviously difficult to combine more than two alleles in the same locus in rice. In addition, there is still some apprehension about linkage drag, which is more laborious, requiring several backcrosses with large populations for screening (Wang et al. 2015; Khanna et al. 2015; Xiao et al. 2017). Alternatively, transgenesis has been widely adopted to bring a specific gene into crops to improve or impart favorable traits (Ricroch and Henard-Damave, 2016). For example, introducing *phytoene synthase* in combination with *E. uredovora carotene desaturase I* into rice resulted in increased provitamin A content in rice grain, improving its nutritional value (Paine et al. 2005). For disease resistance, *Xa21*, a bacterial blight resistance gene, was transferred into five rice varieties and the transgenic plants exhibited a high resistance to bacterial blight (Zhai et al. 2000).

Global gene expression profiles of a series of transgenic and conventionally bred wheat lines expressing additional genes encoding high molecular weight subunits of glutenin showed that presence of the transgenes does not significantly alter gene expression. The transgenic plants could thus be considered substantially equivalent to untransformed parental lines (Baudo et al. 2006). Comparing the transgenic plants expressing the bacterial blight resistance gene *Xa21* with the MAS-introduced equivalent lines with the resistance gene also demonstrated that both were indeed almost the same at molecular level, and most pathways perturbed by transgenesis were also disturbed by MAS breeding (Gao et al. 2013). In addition, transgenic rice with *Pi54* resistance to blast was not affected by its chromosomal position (Arora et al. 2015). Furthermore, stacking *Pi54* and *Pi54rh* showed a higher level of resistance to a set of highly virulent *M. oryzae* isolates collected from different rice-growing regions (Kumari et al. 2017). Pyramiding of *Pi9* together with multiple genes resistant to brown planthopper, bacterial blight and lepidopteran pest in rice showed significantly higher yield with imparted multiple resistances, including the blast (Li et al. 2021). Here, we report pyramiding of three novel *Pi* genes *Pib*, *Pi25* and *Pi54* in rice via an *Agrobacterium*-mediated transgenic approach, providing more information for effectively breeding rice cultivars with durable blast resistance.

## Materials and Methods

### Development of the *Pi* Gene Fusion Construct with *Pib*, *Pi25* and *Pi54*

The construct in this study was built based on Golden Braid system (Sarrion-Perdigones et al. 2013). The coding DNA fragments of *Pib* (GenBank: AB013448.1), *Pi54* (GenBank: AY914077.1) and *Pi25* (GenBank: HM448480.1) were amplified via PCR method using the specific oligo primers (Table S1A) and then cloned into entry vector pUPD2. Other elements in the construct for the *Pi* gene expression in plants, such as the Pepc promoter and Nos terminater, were also cloned into pUPD2. All the cloning DNA fragments were designed with a flanking *Bsm*BI site. The expression vector pDGB3α2 has two *Bsa*I sites compatible with the cloned fragment in the entry clone. All the cassettes were finally assembled by *Bsa*I and *Bsm*BI digestion and T4 DNA ligase ligation to form the construct pDGB3_alpha2-Pib-Pi25-Pi54 (Fig. S1).

### Generation of Transgenic Rice Plants

The construct with genes *Pib*, *Pi25* and *Pi54* was transferred into *A. tumefaciens* strain LBA 4404 using freeze–thaw method (An et al. 1988). Scutellar calli derived from matured seeds of Kasalath and Zhenghan 10 were used for genetic transformation in *Agrobacterium*-mediated method as described in previous papers (for Kasalath, Saika and Toki, 2010; for Zhenghan 10, Sahoo et al. 2011). The subsequent selection of positive transgenic cells and regeneration of plants were performed on Murashige and Skoog medium with hygromycin. After hardening, plants were grown in a 16/8 h light/dark greenhouse or natural fields.

### Identification of Putative Transgenic Plants

The positively transformed plants were identified by hygromycin tests and PCR methods for *hygromycin phosphotransferase (Hpt)* gene and the *Pi* transgenes. Genomic DNA was extracted from young seedling leaves using the cetyltrimethylammonium bromide method (Murray and Thompson 1980). The PCR primers were shown in Table S1B and PCR amplification was conducted as required for the specific PCR primers used. The amplified fragments were validated by Sanger sequencing. For analysis of the *Pi* transgene expression, reverse transcription PCR (RT-PCR) was conducted. Total RNA from fresh leaves was isolated according to the manufacturer’s manual using RNAiso Plus (Takara, Dalian, China). First-strand cDNA synthesis was performed using a PrimeScript First-Strand cDNA Synthesis Kit (Takara, Dalian, China) according to the manufacturer’s instructions. The PCR primers (Table S1C) were designed based on the promoter Pepc and the *Pi* transgene sequences.

### Analysis of Indigenous Homologous Alleles of *Pib*, *Pi25* and *Pi54*

The indigenous homologous alleles of *Pib*, *Pi25* and *Pi54* in both Kasalath and Zhenghan 10 were amplified from the genomic DNA and/or cDNA using PCR methods, and the amplified DNA fragments were sequenced using the Sanger method. Genomic DNA and/or cDNA were obtained as described above. The PCR primers (Table S1D) were designed on reported sequences in literatures to differentially amplify the homologous alleles. Expressions of the indigenous genes were analyzed via RT-PCR using the specific primers (Table S1C) as described above for analysis of the *Pi* transgene expressions.

### Phenotyping for Resistance to *M. oryzae*

The transgenic lines with the untransformed parents were grown in 60 cm × 30 cm × 4 cm plastic trays with sieved garden soil. For the inoculation experiment, three-week-old rice seedlings with 3–4 leaves were placed in an inoculation chamber and inoculated by spraying with blast spore suspension (~ 5 × 10^− 5^ spores per milliliter) with 0.02% Tween-20. A total of 15 blast isolates of the fungus *M. oryzae* were collected widely in rice-growing areas in China, including several from Sichuan Province of China (Yang et al. 2013). Inoculated seedlings were kept in dark at 27 °C with 95–100% relative humidity for 24 h and then transferred to greenhouse where they were grown under a 12 h light/12 h dark cycle and about 90% relative humidity for 7 days to allow disease development.

The transgenic plants were also planted in the fields to evaluate the disease resistance under natural infection. The transgenic and corresponding nontransgenic lines were grown with universally susceptible rice Lijiangxintuanhegu side by side in row in Chengdu (103°25′ E, 30°38′ N). Fifty plants per line were arranged in 10 plants per row spaced at 18 cm × 25 cm. Three replicates were conducted in a random arrangement of all the test lines.

### Analysis of Morphological Traits

To evaluate agronomic performance of the transgenic rice, a field experiment was performed in Chengdu (104°11′ E, 31°97′ N) during the summer season. Seeds used for the experiment came from the same lot used for leaf blast resistance evaluation and three independent transgenic lines with its untransformed plants for both Kasalath and Zhenghan 10 were tested in the experiment. One hundred plants per line were grown to maturity in 10-row plots spaced at 18 cm × 25 cm with three replicates in a random arrangement of all the test lines. Data on yield components including heading date, plant height, tillers per plant, panicle length, grains per panicle, seed-setting rate, and 100-grain weight were recorded from five or more plants per line. The heading date was calculated as days from seeding to 50% of the plants flowered. Seeds were air-dried in glasshouse and then weighed.

### RNA-Seq and Transcriptome Analysis

The RNA-sequencing (RNA-Seq) was performed on an Illumina platform (BioMarker, Beijing, China) and paired-end reads were generated for transcriptome analysis. Total RNA from 10 young seedlings with 3–4 leaves for both the transgenic and untransformed lines was isolated by using TRIzol reagent (Invitrogen, CA, USA) according to the manufacturer’s instructions. The RNA concentration and purity were measured using a NanoDrop 2000 (Thermo Fisher Scientific, DE, USA). RNA integrity was assessed using RNA Nano 6000 Assay Kit of Agilent Bioanalyzer 2100 system (Agilent Technologies, CA, USA). A total amount of 1 μg RNA per sample was used as input material for sequencing library. The library was generated using NEBNext UltraTM RNA Library Prep Kit for Illumina (NEB, MA, USA) following manufacturer’s recommendations and index codes were added to attribute sequences to each sample. The library quality was assessed on an Agilent Bioanalyzer 2100 system.

The obtained raw sequences were first transformed into clean reads after data processing. These clean reads were then mapped to the reference genome sequence of Nipponbare (MSU7.0). Only reads with a perfect match or one mismatch were further analyzed and annotated based on the reference genome via Hisat2 tools software (Kim et al. 2015). Gene function was annotated based on the following databases: Nr (NCBI nonredundant protein sequences); Nt (NCBI nonredundant nucleotide sequences); Pfam (Protein family); KOG/COG (Clusters of Orthologous Groups of proteins); Swiss-Prot (a manually annotated and reviewed protein sequence database); KO (KEGG Ortholog database); and GO (Gene Ontology). Gene expression levels were estimated by fragments per kilobase of transcript per million fragments (FPKM) mapped. Differential expression analysis between transgenic and nontransgenic samples in three replicates for both Kasalath and Zhenghan 10 was performed using the DESeq2 (Love et al. 2014). Statistical analysis of the differential gene expression was based on a model of negative binomial distribution, and the resulted *P*-values were adjusted using the Benjamini and Hochberg’s approach for controlling the false discovery rate. Genes with an adjusted P-value < 0.05 were assigned as differentially expressed genes (DEGs).

### Quantitative Real-Time PCR Analysis

Quantitative real-time PCR (qRT-PCR) was conducted using total RNA isolated as described above for RNA-Seq. Nucleotide sequences of the selected genes for qRT-PCR were downloaded from the rice database (http://rice.plantbiology.msu.edu) and used for primer design. All the primer pairs were listed in Table S1E and Table S1F. PCR amplification was conducted with ChamQ Universal SYBR qPCR Master Mix (Vazyme, Nanjing, China) on a Roche Light Cycler 96 according to the manufacturer’s instructions. Ubiquitin gene (LOC_Os03g13170) was used as internal control. The 2^-△Ct^ method was used to analyze relative transcript levels of gene expression.

## Results

### Genetic Transformation and Molecular Analysis of Transgenic Plants

**Fig. 1:**
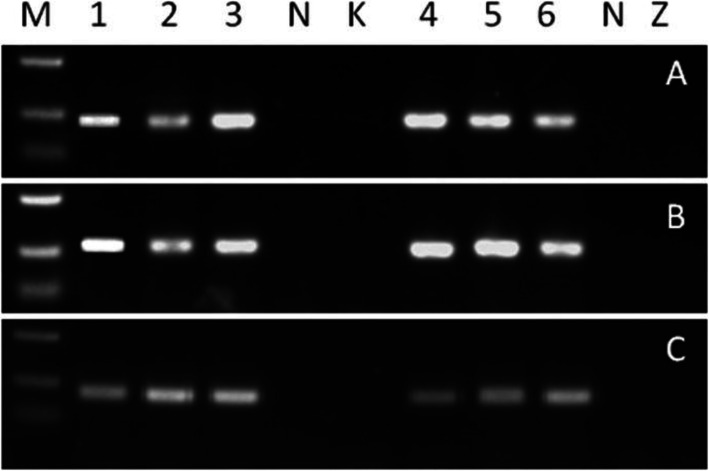
Expression levels of the *Pi* transgenes of *Pib* (A), *Pi25* (B) and *Pi54* (C) in Kasalath and Zhenghan 10 detected by RT-PCR. Line 1, 2and 3 are the transgenic lines of Kasalath. Line 4, 5 and 6 are the transgenic lines of Zhenghan 10. K: nontransgenic Kasalath; Z: nontransgenic Zhenghan 10. N: negative control; M: molecular marker (500, 250 and 100 bp)

Two rice varieties, Kasalath and Zhenghan 10, were transformed with *A. tumefaciens* LBA4404 containing the plasmid pDGB3_alpha2-Pib-Pi25-Pi54. *Pib* is identical to the sequence reported in rice Tohoku IL19 (Wang et al. 1999). *Pi54* is the same gene from Tetep (Sharma et al. 2010), and *Pi25* is the *Pid3* allele cloned from Gumei2 (Chen et al. 2011). The regenerated plants (T_0_) were screened by PCR methods for presence of the marker gene *Hpt* and the *Pi* transgenes of *Pib*, *Pi54* and *Pi25* so that the T-DNA integrated into the genomes of Kasalath and Zhenghan 10 was assured. The selected positive plants were further analyzed by RT-PCR to ensure expression of the *Pi* transgenes in the transgenic plants. Independently derived T_0_ plants with expression of the *Pi* transgenes were selected. These plants were self-pollinated to produce T_1_ plants, and so on to obtain the homozygous lines in T_3_ generation. Three independent transgenic lines with stable expression of the transgenes (Fig. 1) were used in further studies to evaluate the disease resistance and agronomic traits.

In order to clarify the roles of *Pi* transgenes in the transgenic plants, it is necessary first to ascertain the indigenous alleles of *Pib*, *Pi54* and *Pi25* in both Kasalath and Zhenghan 10. PCR was used to amplify the genes from the genomic DNA and/or cDNA, and the amplified fragment was then sequenced. In Kasalath, *Pib* (GenBank No.MW194251) is a nonfunctional allele with a premature stop codon in the first exon; *Pi25* (GenBank No. MW194249) is a functional allele assigned as *Pid3*-*I1* (Lv et al. 2017); *Pi54* (GenBank No. MW194246) is also a pseudogene, which is exactly the same as the allele in Nipponbare genome (Kumari et al. 2013). In Zhenghan 10, *Pib* (GenBank No. MW194250) is a functional allele assigned as *Pib_40286*, which is similar to the functional *Pib_Engkatek* (Vasudevan et al. 2016); *Pi25* (GenBank No. MW194243) is a susceptible allele with a premature stop codon as the allele in Nipponbare (Chen et al. 2011), and *Pi54* (GenBank No. MW194245) is an allele with only two amino acids different from that in Tetep genome (Rai et al. 2011).

**Fig. 2:**
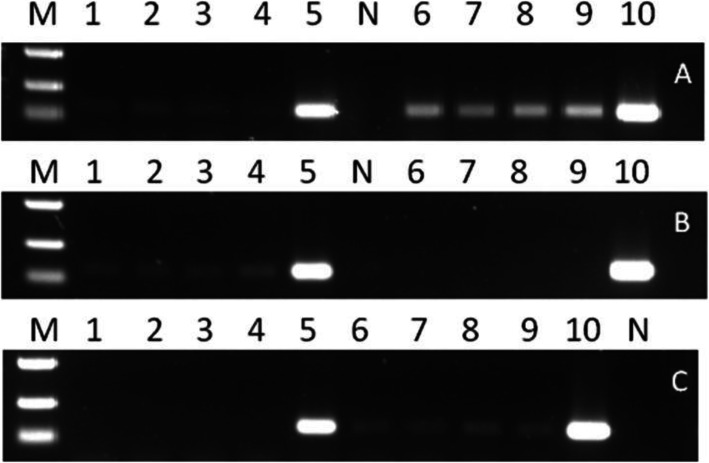
Expression levels of the indigenous homologue genes of *Pib* (A), *Pi25* (B) and *Pi54* (C) in Kasalath and Zhenghan 10 detected by RT-PCR. Line 1, 2 and 3 are the transgenic lines of Kasalath. Line 4 and 5 are nontransgeneic Kasalath cDNA and genomic DNA, respectively. Line 6, 7 and 8 are transgeneic lines of Zhenghan 10. Line 9 and 10 are nontransgenic Zhenghan 10 cDNA and genomic DNA, respectively. N: negative control; M: molecular marker (500, 250 and 100 bp)

Transcriptions of the indigenous homologous alleles were further examined by RT-PCR (Fig. 2). For Kasalath, the indigenous alleles of *Pib* and *Pi25* were expressed in quite low levels, but expression of the *Pi54* allele could not be detected. For Zhenghan 10, the observed expression was also quite low for the *Pib* and *Pi54* alleles, but the *Pi25* allele seemed to have no expression at all. The nonfunctional *Pi* pseudogenes in Kasalath (*Pi54*) and Zhenghan 10 (*Pi25*) had lost their transcriptional abilities but the *Pib* allele in Kasalath was expressed, The observed expression of the functional alleles are consistent with reports before (Wang et al. 2001; Shang et al. 2009; Rai et al. 2011), indicating that the *Pi* genes in both cultivars are constitutively expressed at low levels.

### Performance of the Transgenic Plants in Response to *M. oryzae* Infection

**Fig. 3:**
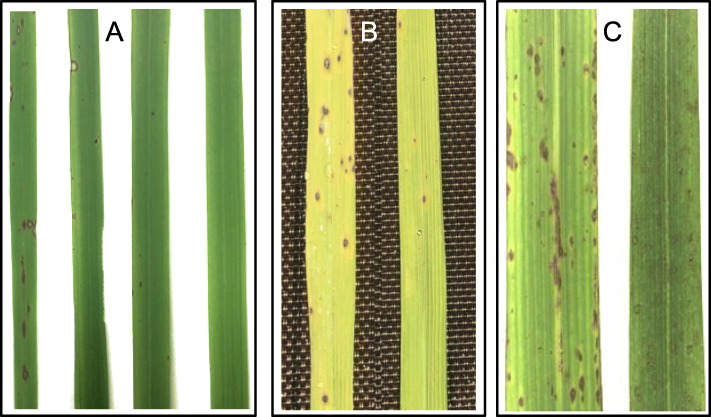
Four types of disease symptoms (A) on the leaves observed in greenhouse from left to right: susceptible (S), partial susceptible (MS), partial resistant (MR) and resistant (R). Blast lesions on the leaves observed in natural field for Kasalath (B) and Zhenghan 10 (C); right is the transgenic, and left is the nontransgenic

To evaluate the disease resistance of the transgenic plants stacked with the three genes *Pib*, *Pi25* and *Pi54*, 15 isolates of *M. oryzae* were used in a greenhouse experiment, in which 11 strains were collected widely from rice-growing areas of China in 2008–2009 (Yang et al. 2013) and additional 4 were isolated recently from rice fields in Sichuan Province of China. The disease reaction was recorded at seven days after inoculation, and leaf lesion types (Fig. 3A) were scored as resistance (R), medium resistance (MR), medium susceptibility (MS), and susceptibility (S). The results (Table 1) showed that two transgenic lines, TL-1 and TL-2 for both Kasalath and Zhenghan 10, consistently exhibited some improvements in resistance to 4 and 3 isolates of the pathogen, respectively, in which 2 strains are common for both Kasalath and Zhenghan 10.

**Table 1:** Rice blast resistance spectra of the transgenic rice^*^

Isolate	rice lines					
	KSL-N	KSL-T1	KSL-T2	ZH-N	ZH-T1	ZH-T2
AH1	R	R	R	R	R	R
CQ1	MS	MS	MS	R	R	R
CQ2	R	R	R	R	R	R
GD1	S	R	R	R	R	R
HB1	S	MR	MR	S	MS	MS
JS1	MR	MR	MR	MS	MS	MS
JS2	R	R	R	R	R	R
LN1	S	R	R	S	R	R
SC2	MR	MR	MR	MR	MR	MR
YN1	R	R	R	R	R	R
YN2	S	MS	MS	R	R	R
I03	R	R	R	S	R	R
I08	MR	MR	MR	R	R	R
J02	R	R	R	R	R	R
J05	S	S	S	S	S	S

*R: resistance, MR: medium resistance, MS: medium susceptibility, S: susceptibility; KSL-N: Kasalath nontransgenic line; KSL-T: Kasalath transgenic line; ZH-N: Zhenghan 10 nontransgenic line; ZH-T: Zhenghan 10 transgenic line

The transgenic plants were also planted in the field to evaluate the disease resistance by natural infection in Chengdu where blast disease occurs in rice-growing season almost every year. The three transgenic lines with the untransformed parent for each of the tested cultivars were grown in the field. Imparted resistances for Kasalath and Zhenghan 10 were also observed in the leaves in 2019 and in 2020, respectively (Fig. 3B and C).

### Morphological Features and Agronomic Traits of the Transgenic Plants

**Fig. 4:**
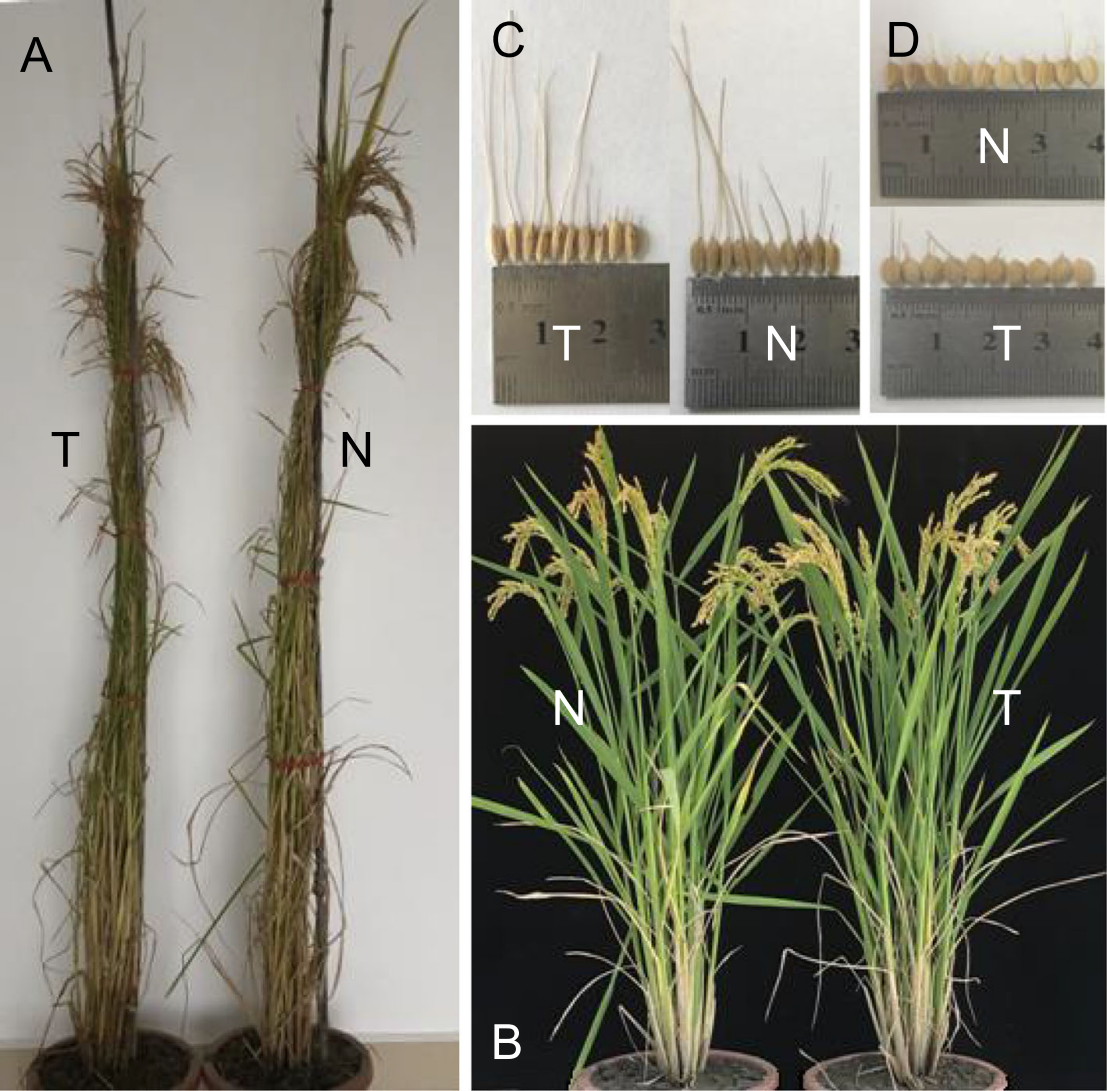
Phenotypes of the transgenic plants of Kasalath and Zhenghan 10. A: Kasalath plants; B: Zhenghan 10 plants; C: Kasalath seeds; D: Zhenghan 10 seeds. T: Transgenic; N: Nontransgenic

**Fig. 5:**
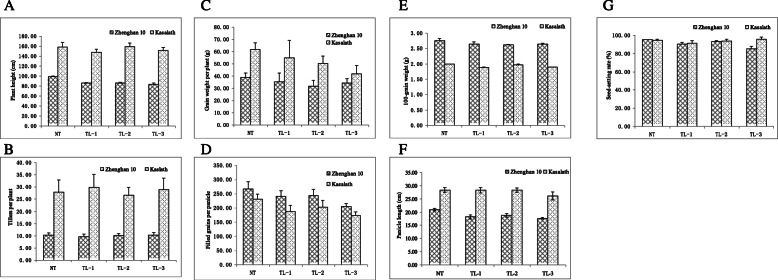
Main agronomic traits of the transgenic plants of Kasalath and Zhenghan 10. Plant height; B: Teller numbers per plant; C: Grain weight per plant; D: Filled grain number per panicle; E: 100-grain weight; F: Panicle length; G: seed-setting rate. Error bars showing the SD based on three replicates

The transgenic lines were planted in Chengdu, where no blast disease typically occurs in the growing season. Differences of growth and development among the three transgenic lines of both Kasalath and Zhenghan 10 with their nontransgenic parents were observed during the growth period. Heading dates for Kasalath and Zhenghan 10 were 102 and 90 days after seeding, respectively, and 2–4 days less for transgenic plants of both cultivars. At time of maturity several primary morphological features and agronomic traits were further measured. The transgenic plants were shorter in both cultivars, particularly for Zhenghan 10 (Fig. 4A, B and 5A), whereas tiller numbers per plant (Fig. 5B) did not differ between the transgenic and nontransgenic lines in either cultivar. Decreased grain yield per plant (Fig. 5C) in the transgenic lines was observed in both Kasalath and Zhenghan 10, which mainly resulted from reduced filled grains per panicle (Fig. 5D) as grain size and weight (Fig. 4C, D and 5E) showed almost no change between the transgenic and nontransgenic lines in either cultivars. Panicle length (Fig. 5F) was slightly shorter in transgenic lines, and this reduction was particularly true in Zhenghan 10. There was not clear difference in seed-setting rates (Fig. 5G) between transgenic and nontransgenic lines for either Kasalath or Zhenghan 10 except transgenic line TL-3 of Zhenghan 10.

### Transcriptomic Profiles and Pathways Influenced by the *Pi* Transgenes

**Fig. 6:**
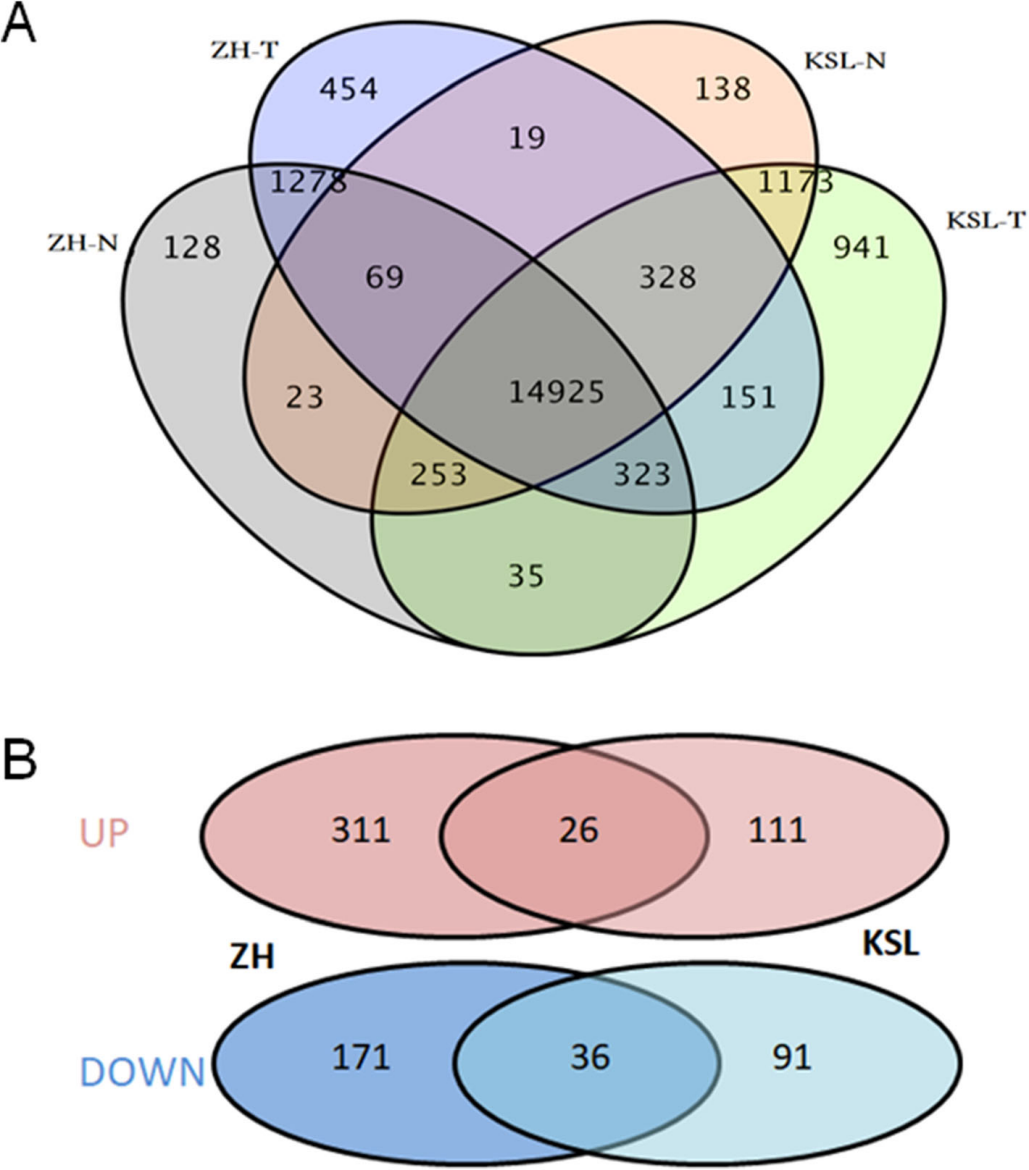
Differential transcription in the transgenic plants of Kasalath and Zhenghan 10 . A, Venn diagram of expressed genes in the transgenic lines and nontransgenic lines. B, Venn diagram of DEGs in the transgenic lines. KSL-T: transgenic Kasalath; KSL-N: nontransgenic Kasalath; ZH-T: transgenic Zhenghan10; ZH-N: nontransgenic Zhenghan10. UP: upregulated genes; DOWN: downregulated gens

To further investigate the effects of the *Pi* transgenes, an RNA-seq experiment with three replicate samples of a transgenic line for both Kasalath and Zhenghan10 was conducted. Global gene transcription analysis (Fig. 6A) revealed that majority of the expressed genes (more than 90%) were expressed in both transgenic and wild-type plants for both cultivars. The exclusively expressed genes were observed in both transgenic and nontransgenic plants, The numbers expressed only in the transgenic plants were 8.0% and 5.4% whereas the numbers of expressed solely in the nontransgenic plants were 1.5% and 2.6% for Kasalath and Zhenghan 10, respectively. These results indicated that the *Pi* transgenes in both rice cultivars had some impact on global gene transcription. Inducing gene expression was dominant, but inhibition was also a mechanism to regulate gene transcription. The effect on gene expression also exhibited some variations between the two varieties. Following global analysis of the expressed genes, 264 and 544 differentially expressed genes (DEGs, Fig. 6B) were further identified in Kasalath (Table S2) and Zhenghan 10 (Table S3), respectively. More upregulated genes, 137 and 337 respectively for Kasalath and Zhenghan 10, were observed, which further indicates that enhancing gene transcription was more than inhibiting in the transgenic plants. To validate the RNA-Seq results, 9 genes randomly selected from the DEGs (Table S1 E) were further analyzed by qRT-PCR method. The detected transcript levels of the selected genes are basically consistent with those detected in the RNA-Seq experiment even though there are some differences between the two methods (Fig. S2).

The DEGs were then annotated based on 26 categories of COG (Cluster of Orthologous Groups) database classification (Tatusov et al. 2000) resulting in 106 genes assigned to 20 categories for Kasalath (Fig. S3A) and 216 genes assigned to 22 categories for Zhanghan 10 (Fig. S3B). The overall distribution of the genes was similar between two cultivars. For Kasalath the T group of signal transduction mechanisms was the largest, which also ranked third in Zhenghan 10. However, for Zhenghan 10 the Q group of secondary metabolite biosynthesis, transport and catabolism containing 24 genes was the largest group, which also contained 8 genes in Kasalath. Interestingly, the V group of defense mechanisms and the K group of transcription contained many genes in both cultivars. All these findings might imply that an improved immune response occurred in the transgenic plants of both cultivars.

The DEGs were further annotated using Kyoto Encyclopedia of Genes and Genomes (KEGG) pathways (Kanehisa et al. 2004). In total 36 genes in Kasalath were assigned to 30 pathways (Fig. S4A), whereas 64 genes in Zhenghan 10 were allocated to 60 pathways (Fig. S4B). Four pathways appeared in both cultivars: 1) plant hormone signal transduction in the category of environmental information processing, 2) plant-pathogen interaction and circadian rhythm-plant in organismal systems, 3) carotenoid biosynthesis and 4) glycerolipid metabolism in metabolism. These results suggest that plant growth, disease resistance and developmental procedures might be affected by the *Pi* transgenes. In particular, carbon metabolism pathway regulated in both cultivars might suggest the energy expenditure required to enhance the disease resistance. In addition, many different pathways regulated between two cultivars were observed. Thus, different responses based on different genetic backgrounds occurred, which was also reported in previous research on *Magnaporthe oryzae* infection (Tian et al. 2018).

The distinct response pathways in the transgenic plants were further confirmed by KEGG pathway enrichment analysis of the DEGs. Fig. S5A and B display the top 20 pathways with the lowest q-values in Kasalath and Zhenghan 10, respectively. In Kasalath three most significantly regulated pathways were circadian rhythm, nitrogen metabolism and glycerolipid metabolism. However in Zhenghan 10, phenylalanine metabolism, plant hormone signal transduction and zeatin biosynthesis were the three pathways that were most significantly regulated. Pathways such as glycerolipid metabolism, carotenoid biosynthesis and phenylpropanoid biosynthesis were significantly regulated in both cultivars. These results indicated the regulation of defense-related primary and secondary metabolic pathways, as reported for *Pi54* (Gupta et al. 2012) and in the context of blast pathogen infection (Tian et al. 2018).

The T-DNA insertion in genome might impact gene expression and the transcriptome (Hsing et al. 2007). To justify the RNA-seq results, a quantitative real-time PCR was further conducted using three independent transgenic lines including the one used in RNA–seq. Eleven DEGs including eight downregulated and three upregulated, from the significantly pathways in the above KEGG pathway enrichment analysis in Kasalath or Zhenghan 10 or both were selected in the experiment (Table 2). *NPR1* (Yuan et al. 2007) is a functionally conserved gene in diverse plant species in disease resistance of salicylic acid pathway so that *OsNPR1* (LOC_Os01g09800) is particularly selected in the experiment. Among the twelve selected genes the downregulated and the upregulated genes were basically also downregulated and upregulated respectively in Kasalath (Table S4) and Zhenghan 10 (Table S5) in the PCR experiment. However, a few inconsistencies were observed as well. For an example, downregulated gene LOC_Os04g40040 in RNA-seq study were almost indifferent among three transgenic lines and their parent or even upregulated in the PCR experiment in both cultivars. *OsNPR1*, not significantly regulated in either Kasalath or Zhenghan 10 in the RNA-Seq analysis, had some variations among wild-type and transgenic lines in both cultivars. Such casual variations might not be resulted from the *Pi* transgenes, and the expressions of the *NPR1* would not support the improved disease resistance from regulation of the salicylic acid pathway.

**Table 2:**
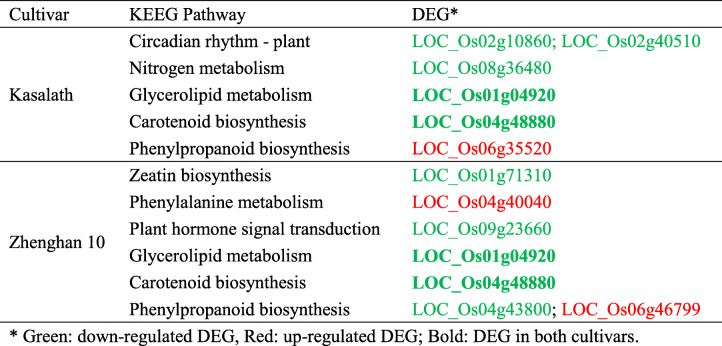
Genes selected from DEGs for the qRT-PCR to be analyzed in three independent transgenic lines of both cultivars

*Green: down-regulated DEG, Red: up-regulated DEG; Bold: DEG in both cultivars

## Discussion

Creating rice cultivars with durable disease resistance against multiple *Magnaporthe oryzae* strains by stacking *Pi* genes is of high interest among breeding scientists. MAS is a well developed method to stack multiple genes, and pyramiding of *Pi* genes based on this technology has been used in both *indica* and *japonica* rice in various breeding programs (Fukuoka et al. 2015; Orasen et al. 2020; Xiao et al. 2020). Combination of *Pi* genes even with other *R* genes to bacterial blight, and sheath blight has been achieved in resistance to multiple diseases (Ramalingam et al. 2020).

Rice with gathered *Pi* genes through MAS approach shows enhanced blast resistance, but the resistance levels are sometimes lower than what could be expected with the individual *R* genes together. Among *Pi1*, *Piz-5* and *Pita*, the resistance spectrum of lines with various gene combinations matched the predictions based on single gene performance (Hittalmani et al. 2000) (Khanna et al. 2015). Three-gene pyramids of *Pi54* + *Pi1* + *Pita*, *Pi9* + *Pib* + *Pi5* and *Pi2* + *Pib* + *Pi5* were much more effective than that of two-gene pyramids and monogenic lines in imparting broad-spectrum resistance to diverse isolates of blast pathogen through effective complementation (Khanna et al. 2015). However, Pyramiding of *Pizt* and *Pi54* showed an additive effect on resistance to panicle blast, while lines pyramiding of *Pi9* and *Pi54* exhibited lower resistance levels than *Pi9* monogenic lines (Xiao et al. 2017). Evaluation of 277 rice accessions (Wu et al. 2015) inoculated with 76 *M. oryzae* isolates revealed some differences between *indica* and *japonica* rice. Combinations of *Pi9* + *Pi54*, *Pid3* + *Pigm*, *Pi5* + *Pid3* + *Pigm*, *Pi5* + *Pi54* + *Pid3* + *Pigm*, *Pi5* + *Pid*3 and *Pi5* + *Pit* + *Pid3* were able to confer resistance against the pathogen in the *indica* accessions, whereas in the *japonica* accessions combinations of *Pik* + *Pib*, *Pik* + *Pita*, *Pik* + *Pb1*, *Pizt* + *Pia* and *Pizt* + *Pita* shown effective resistance. Analyzing 9 *Pi* genes *Pi37*, *Pit*, *Pid3*, *Pigm*, *Pi36*, *Pi5*, *Pi54*, *Pikm* and *Pb1* in four varieties (Jiang et al. 2019) demonstrated that single-gene lines with *Pigm* or *Pid3* always exhibited significantly enhanced resistance during the whole growth period relative to their recurrent parents. But, single-gene lines with *Pi37*, *Pi5*, *Pit*, *Pi36*, *Pi54* or *Pikm* showed significantly enhanced resistance only in some of the four varieties, and no obviously enhanced resistance was observed in single-gene line with *Pb1* during the entire growth period. For double-gene lines, most of them showed improved resistance, and the lines with *Pi3 7* + *Pid3*, *Pi5* + *Pi54*, *Pi54* + *Pid3* or *Pigm* + *Pi37* exhibited significantly enhanced resistance with observable additive effects.

There have long been evidences that disease resistance may affect crop performance (Brown 2002). Actually, a genome-wide association study (Wang et al. 2015) revealed that 16 markers from 21 simple sequence repeat markers distributed on rice chromosomes 2 to 12 associated with blast resistance were also associated with seed weight, heading date, and plant height; shorter plants were also significantly correlated with blast resistance, and *Pi-ta* was associated with lighter seed weights. A recent study on pyramiding of *Pi2*, *Pi46* and *Pita* (Xiao et al. 2019) showed that two-gene pyramids and three-gene pyramids exhibited a wider resistance spectrum than the monogenic lines. The reduced grain yields were observed in the three pyramid lines of *Pi2*, *Pi46* and *Pita*, even though the improved plants with the two gene pyramids showed comparable agronomic traits compared with their recurrent parents. The introduced *Pi* genes impacted all the traits related to yield including heading date, panicle length, grains per panicle, 1000-grain weight, plant height, tillers per plant and spikelet fertility. Therefore, the linkage with *Pi* genes from donor plants related to yield traits is always a problem for stacking *Pi* genes in MAS technology. Clearly evidences of such linkage drag have been described more in several *Pi* genes. A fragment with 6.4 Mb around the blast *R* gene *Pi33* from a wild rice was found in IR64 introgression lines (Ballini et al., 2007), and 11.6 Mb of chromosomal fragment around *Pita* from donor (Tetep) was also identified in BC5F2 individuals (Jia 2009). The *pi21* was reported to be separable from a closely linked gene of poor eating quality in an early research (Fukuoka et al., 2009), However it still was found in the pyramided lines at the BC4F3 and BC4F4 generations with the stacked *pi21* reported recently (Angeles-Shim et al. 2020). Nevertheless, success in combining tolerance to multiple biotic and abiotic stresses by pyramiding of seven to ten genes has been also reported recently (Dixit et al. 2020). The pyramided lines were high yielding under various stresses and they also performed well in nonstress conditions without any yield penalty.

Transgenesis is another approach for pyramiding of multiple gene. No such kind of the linked DNA fragment as the linkage drag described above in MAS is introduced into the receptor plants. However, T-DNA insertion in receptor genome could impact nearby gene expression or even block gene transcription if T-DNA is inserted in a functional gene locus (Hsing et al. 2007). However, through careful selection among transgenic lines the T-DNA integrated position has no significant effect on the neighboring genes and their expression (Arora et al. 2015; Magg et al. 2001). The transgenic lines stacked with *Pi54* and *Pi54rh* showed complete resistance to Mo-ei-ger1 strain in comparison to nontransgenic line (Kumari et al. 2018). In this study three *Pi* genes were stacked into both the *indica* and the *japonica* varieties. Enhanced disease resistance was observed as the *Pi* transgenes were integrated into the rice genome and expressed. The adverse influence on plant growth and development, and further reducing grain yield was observed in transgenic lines of both varieties. Without the linkage drag in the transgenic approach yield penalty still occurred to the transgenic plants. The observed early flowering implies shorter vegetable growth period of the transgenic plants, which might be a reason to explain the shorter plant stature and reduced yield. However, the *Pi* gene might play much more important role as revealed in a recent study that knockout of *Pi-ta* susceptible alleles induced drastic fitness decline even in absence of the pathogens (Liu et al. 2020).

*Pi* genes, such as *Pib* (Wang et al. 1999), *Pi25* (Shang et al. 2009), *Pi-ta* (Bryan et al. 2000), *Pi9* (Qu et al. 2006), *Pi2* and *Piz-t* (Zhou et al. 2006), *Pi5* (Lee et al. 2009) and *Pi54* (Rai et al. 2011) are often constitutively expressed in rice plants, and more highly expressed upon infection by the fungus. Higher induced expression is also observed in response to other environmental stimuli (Wang et al. 1999, 2001). Global gene expression by RNA-Seq in this study showed complicated gene expression patterns in both cultivars. Analysis of the DEGs suggested that the *Pi* genes itself could be a major factor impacting growth and development so as to reduce the grain yield. In fact, *Pi* gene locus in rice genome usually is composed of a cluster of NB-LRR protein-coding genes with one functional *R* gene (Liu et al. 2013). Besides, the gene pairs have been discovered recently playing important roles in the disease resistance. In the *Pigm* locus of the rice Gumei 4 (Deng et al. 2017) *PigmR* confers broad-spectrum resistance while *PigmS* suppresses the resistance through competitively attenuation of *PigmR* homodimerization. *PigmS* increases seed production to counteract the yield loss induced by *PigmR*. Such *PigmR* and *PigmS* pair thus balances high disease resistance and yield in rice.

Two NB-LRR protein-coding genes *RGA4* and *RGA5* (Césari et al. 2013), known as sensor and helper, are another kind of the gene pairs, which worked together recognizing the *Magnaporthe oryzae* effector AVR1-CO39 and AVR-Pia. A systemic analysis (Wang et al. 2019) reveals that such NB-LRR pairs of sensor and helper is popular in the rice Tetep genome. The paired sensor and helper are still retained in rice elite cultivars, which are descendants of Tetep, by artificial selection, indicating its important roles in response to the rapidly evolving pathogens (Wang et al. 2019). The sensor is also played essential role in suppressing the helper autoactivation leading to HR-like cell death in absence of the pathogen (Césari et al. 2014), and knocking out the sensor gene in a pair results in a more severe effect than knocking out the helper (Wang et al. 2019). Therefore, stacking or engineering *Pi* genes in such gene pairs or even a functional *R* cluster in transgenesis might be a better way to alleviate the fitness costs associated with the stacked *Pi* genes, and obtain durable resistant rice cultivars.

Clearly, both MAS and transgenesis could reach the goal of stacking *Pi* genes into elite rice and breeding rice cultivars with broad-spectrum resistance to diverse isolates of the pathogen. Transgenic approach is more directly introducing the genes to genome, but it is still required to consider rational utilization of *Pi* genes in combination as suggested for MAS technology (Wu et al. 2015; Xiao et al. 2017, 2020). Particularly the specific mechanism of the *Pi* gene in the disease resistance and the genetic background of receptor cultivar probably need to be paid more attention. Besides, gene editing as a powerful technology has been developed rapidly for crop breeding in these years (Chen et al. 2019). Editing multiple genes using the CRISPR/Cas9 system has got success, including *Pi* genes to improve blast resistance (Li S et al., 2019; Ni et al. 2021; Mishra et al. 2021). Therefore, combination of MAS and transgenesis with gene editing could pave a much broad way towards breeding rice with durable blast resistance.

## Conclusion

In this study, we introduced three *Pi* genes *Pib*, *Pi25* and *Pi54* into the *indica* Kasalath and the *japonica* variety Zhenghan 10. Kasalath is a genotype of the *Pib*-*S* allele, *Pi25*-*R* allele and *Pi54-S* allele, whereas Zhenghan 10 is a genotype of the *Pib*-*R* allele, *Pi25*-*S* allele and *Pi54-R* allele. Improved blast resistance was observed, but yield loss was also occurred as the transgenic plants of both cultivars had smaller panicle. These results support the idea that immunity is often associated with yield penalties. Global transcription analysis showed that overexpression of the *Pi* transgenes impacted several common pathways for both varieties related to growth and development in addition to the pathways for disease resistance response. Pyramiding of *Pi* genes via transgenesis is potentially a promising approach to improve rice resistance to blast disease. However, pleiotropic effects of the *Pi* gene might result in yield loss so that rational combination of *Pi* genes based on genetic background may be important to balance yield and disease resistance.

## Supplementary Information

**Additional file 1 **Fig. S1, Vector for co-expression of three *Pi* genes *Pib*, *Pi25* and *Pi54*. Fig. S2, qRT-PCR validation of the transcription levels of nine selected genes from DEGs detected by RNA–Seq. Fig. S3A: COG diagram of DEGs identified in Kasalath. Fig. S3B: COG diagram of DEGs identified in Zhenghan 10. Fig. S4A: KEGG classification of DEGs identified in Kasalath. Fig. S4B: KEGG classification of DEGs identified in Zhenghan 10. Fig. S5A: KEGG pathway enrichment bubble diagram of DEGs identified in Kasalath. Fig. S5B: KEGG pathway enrichment bubble diagram of DEGs identified in Zhenghan 10.

**Additional file 2 **Table S1, Primers used in this study. A: Primers for constructing the vector; B: Primers for identification of the transgenes in transgenic plants; C: Primers for the *Pi* gene transcription analysis in RT-PCR; D: Primers for PCR amplification of the indigenous homologue alleles; E: Primers for qRT-PCR to validate the RNA-seq data; F: Primers for qRT-PCR of the selected DEGs; Table S2, DEGs identified in Kasalath; Table S3, DEGs identified in Zhenghan 10. Table S4, qRT- PCR of the selected DEGs for Kasalath; Table S5, qRT- PCR of the selected DEGs for Zhenghan 10.

## Data Availability

The vector used in this study will be available upon request. The datasets of RNA-Seq in this study are available from the corresponding author on reasonable request.

## Abbreviations

PCR: polymerase chain reaction
RT-PCR: reverse transcription-PCR
qRT-PCR: quantitative real-time-PCR
MAS: marker-assisted selection
RNA-Seq: RNA-sequencing
R: resistance
MR: medium resistance
MS: medium susceptibility
S: susceptibility
KOG/COG: Clusters of Orthologous Groups of proteins
KEGG: Kyoto Encyclopedia of Genes and Genomes
DEGs: differentially expressed genes
T_0_: regenerated plants from transgenic cells
T_1_: offspring of T_0_
T_2_: offspring of T_1_
T_3_: offspring of T_2_ and so on
KSL: Kasalath
ZH: Zhenghan 10
KSL-T: Transgenic Kasalath
ZH-T: Transgenic Zhenghan 10
KSL-N: Nontransgenic Kasalath
ZH-N: Nontransgenic Zhenghan 10
NT: Nontansgenic plant
TL: Transgenic line
