# Supplementary material for: Characterization and Evaluation of Transgenic Rice Pyramided with the Pi Genes Pib, Pi25 and Pi54
Source: Rice (N Y). 2021 Sep 7;14:78. doi: 10.1186/s12284-021-00512-w (PMC8423957; doi:10.1186/s12284-021-00512-w)
Supplement: Supplementary file 1 — Additional file 1 Fig. S1, Vector for co-expression of three Pi genes Pib, Pi25 and Pi54. Fig. S2, qRT-PCR validation of the transcription levels of nine selected genes from DEGs detected by RNA–Seq. Fig. S3A: COG diagram of DEGs identified in Kasalath. Fig. S3B: COG diagram of DEGs identified in Zhenghan 10. Fig. S4A: KEGG classification of DEGs identified in Kasalath. Fig. S4B: KEGG classification of DEGs identified in Zhenghan 10. Fig. S5A: KEGG pathway enrichment bubble diagram of DEGs identified in Kasalath. Fig. S5B: KEGG pathway enrichment bubble diagram of DEGs identified in Zhenghan 10. [file 12284_2021_512_MOESM1_ESM.doc]

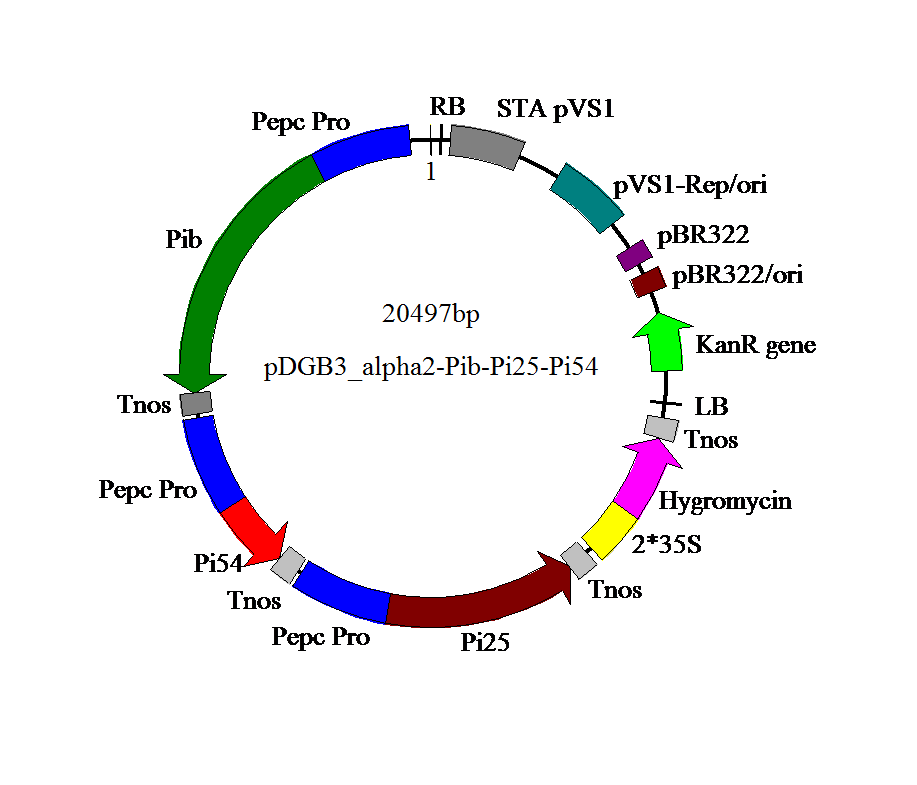


Fig. S1: Vector for coexpression of three *Pi* genes *Pib*, *Pi25* and *Pi54*


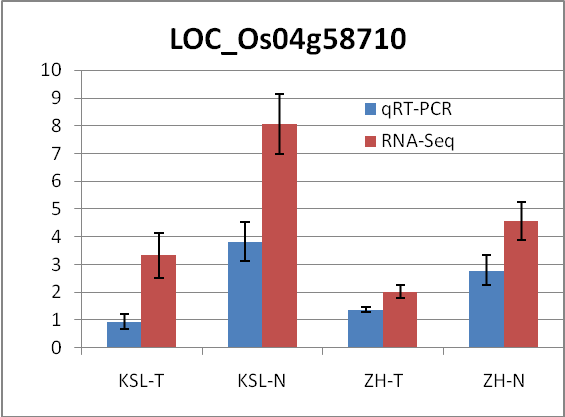


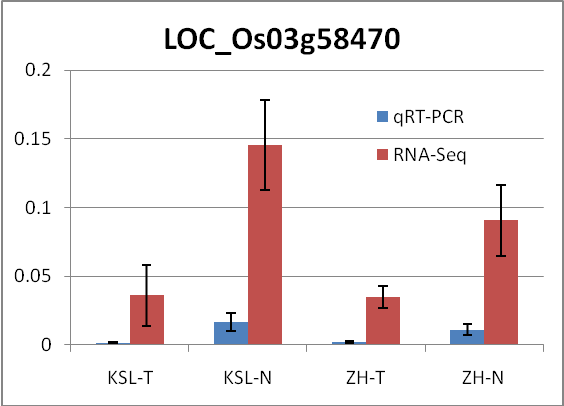


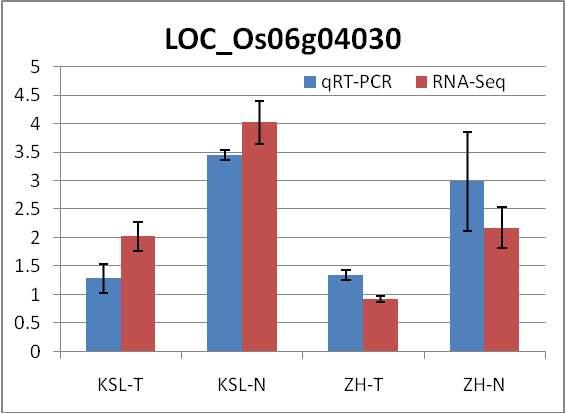


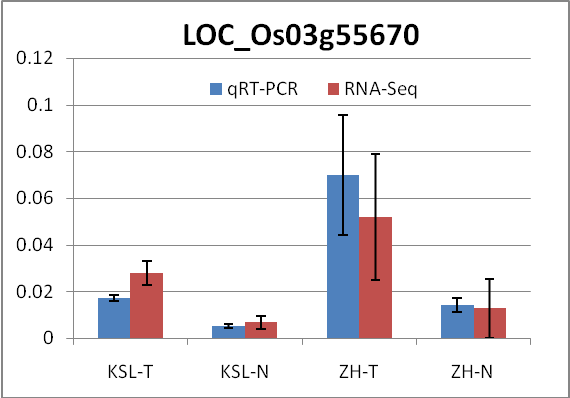


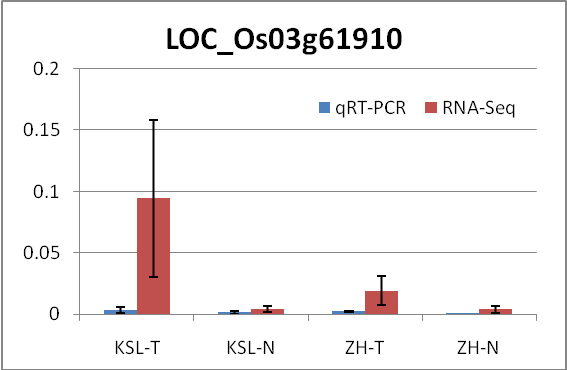


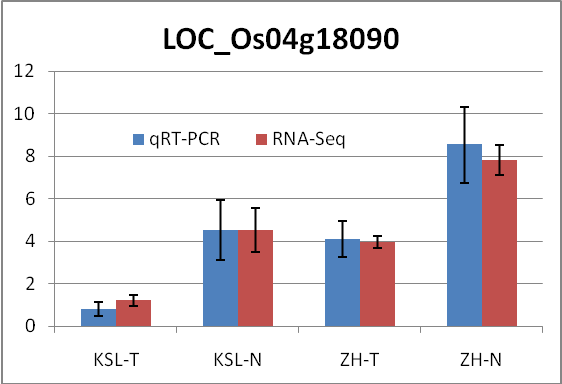


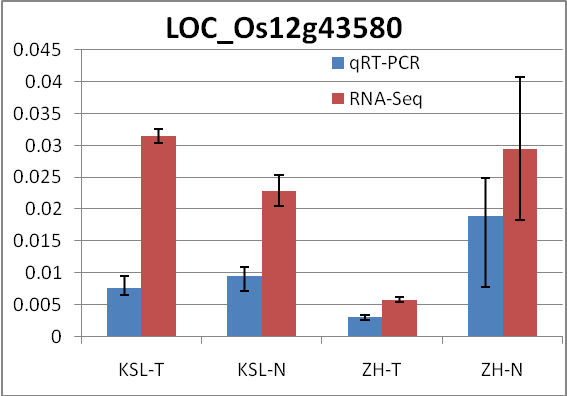


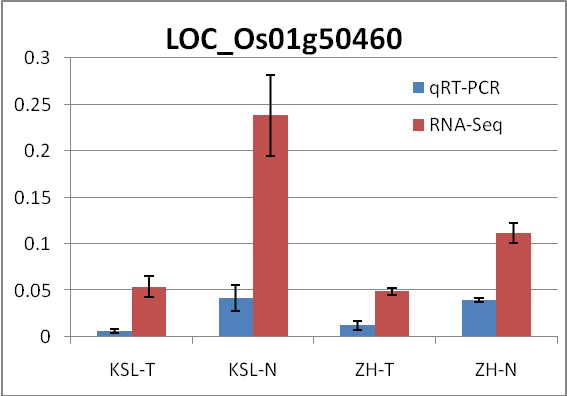


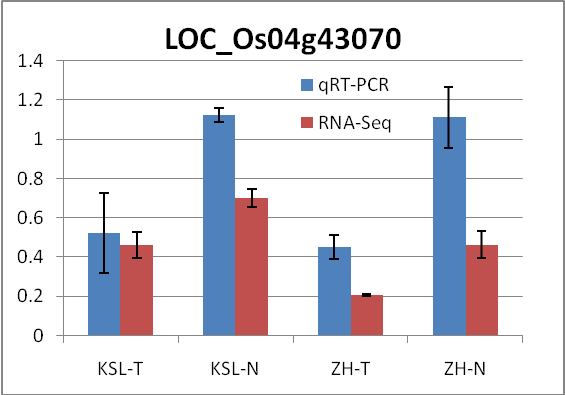


Fig. S2: qRT-PCR validation of the transcription levels of nine selected genes from DEGs detected by RNA–Seq. The number in vertical axis is a relative numeral value to represent transcript levels detected in qRT-PCR and RNA-seq. Error bars showing the SD based on three replicates.


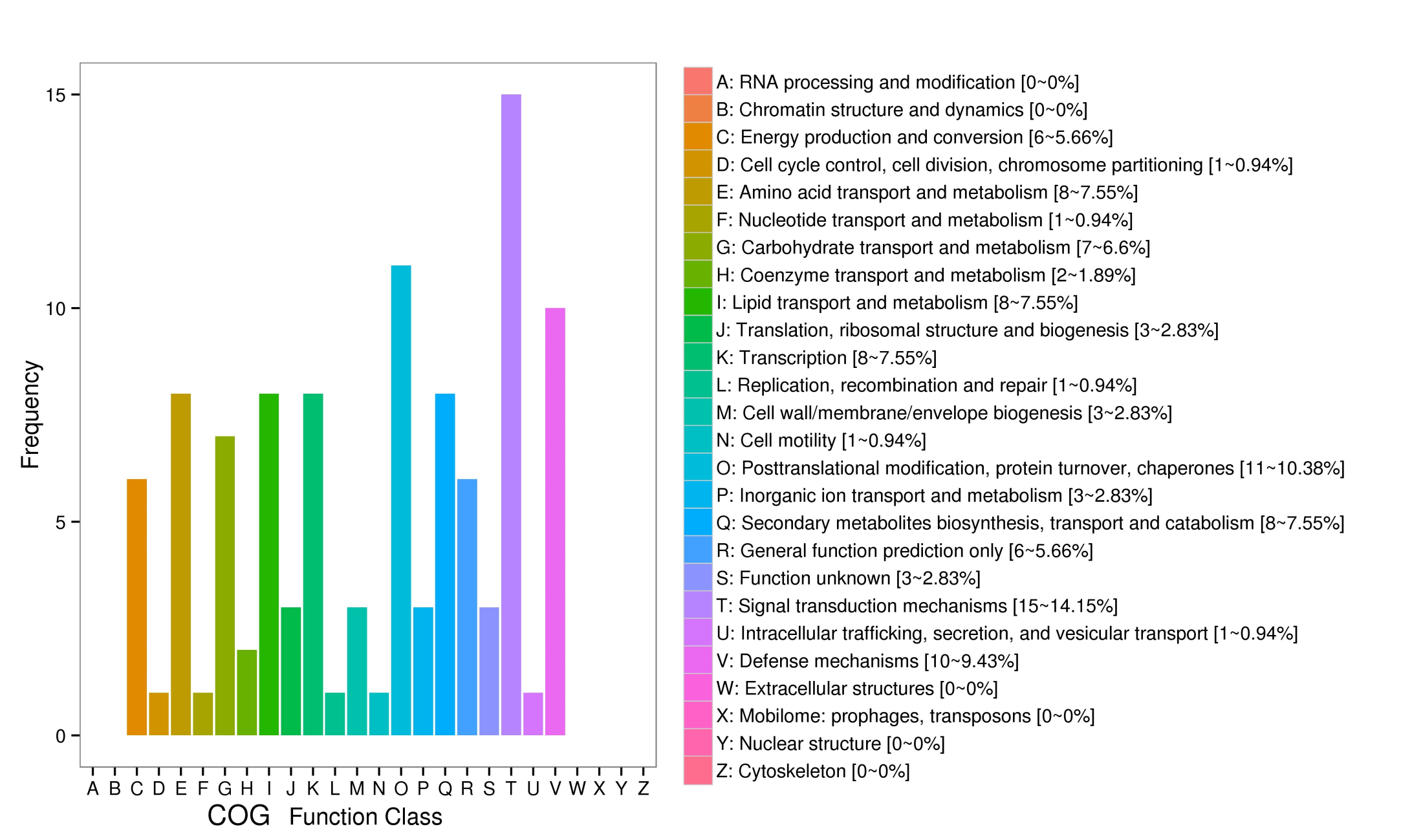


Fig. S3A: COG diagram of DEGs identified in Kasalath. Abscissa: categories of COG. Ordinate: number of genes.


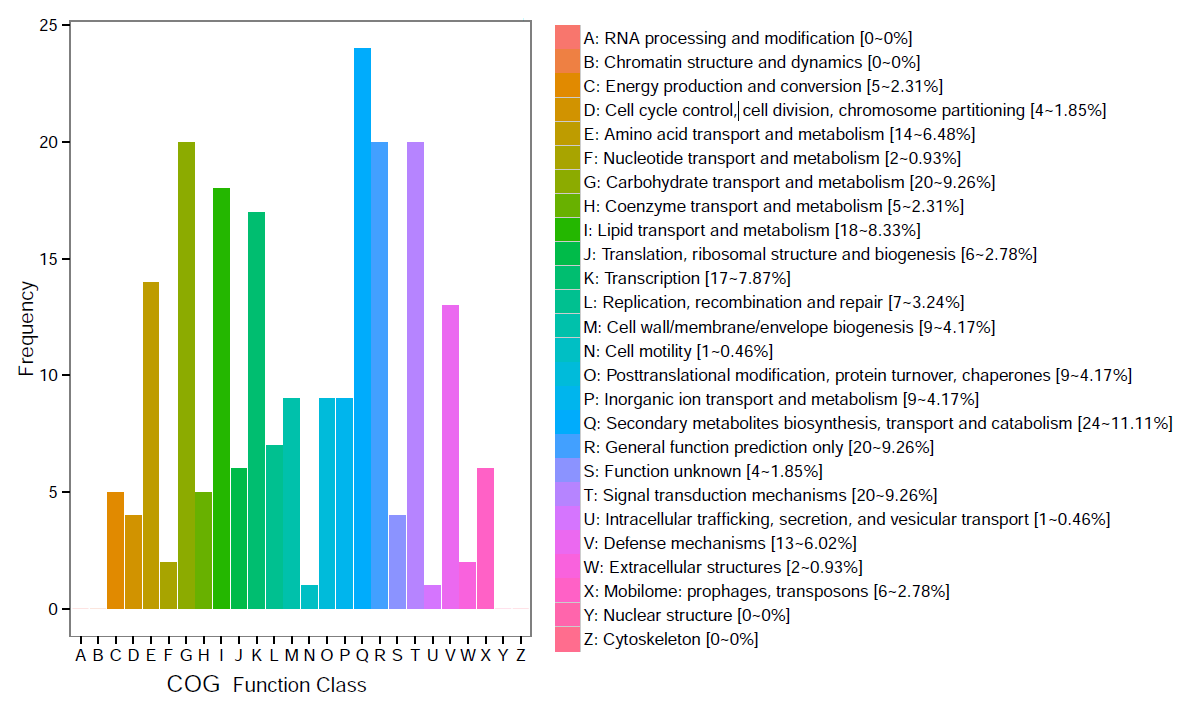


Fig. S3B: COG diagram of DEGs identified in Zhenghan 10. Abscissa: categories of COG. Ordinate: number of genes.


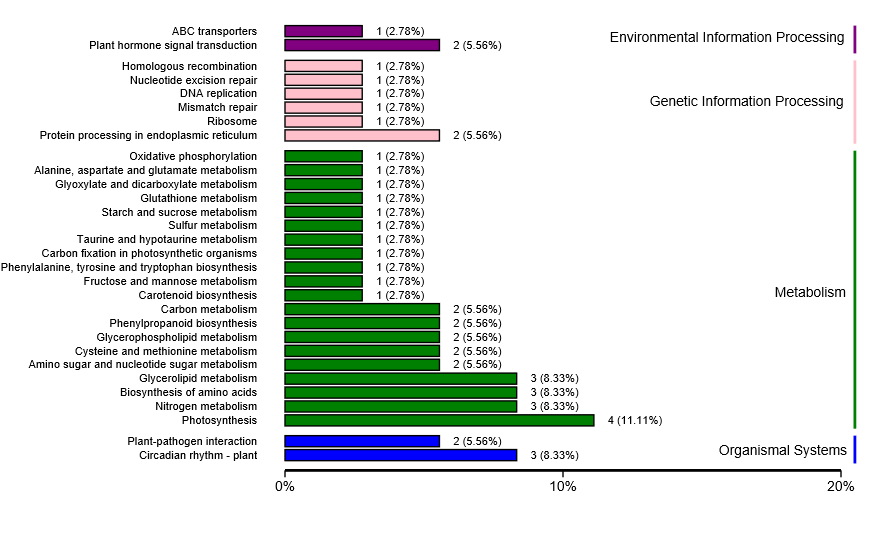


Fig. S4A: KEGG classification of DEGs identified in Kasalath. Vertical axis: KEGG pathway. Horizontal axis: number of genes and proportion of genes in total annotated genes.


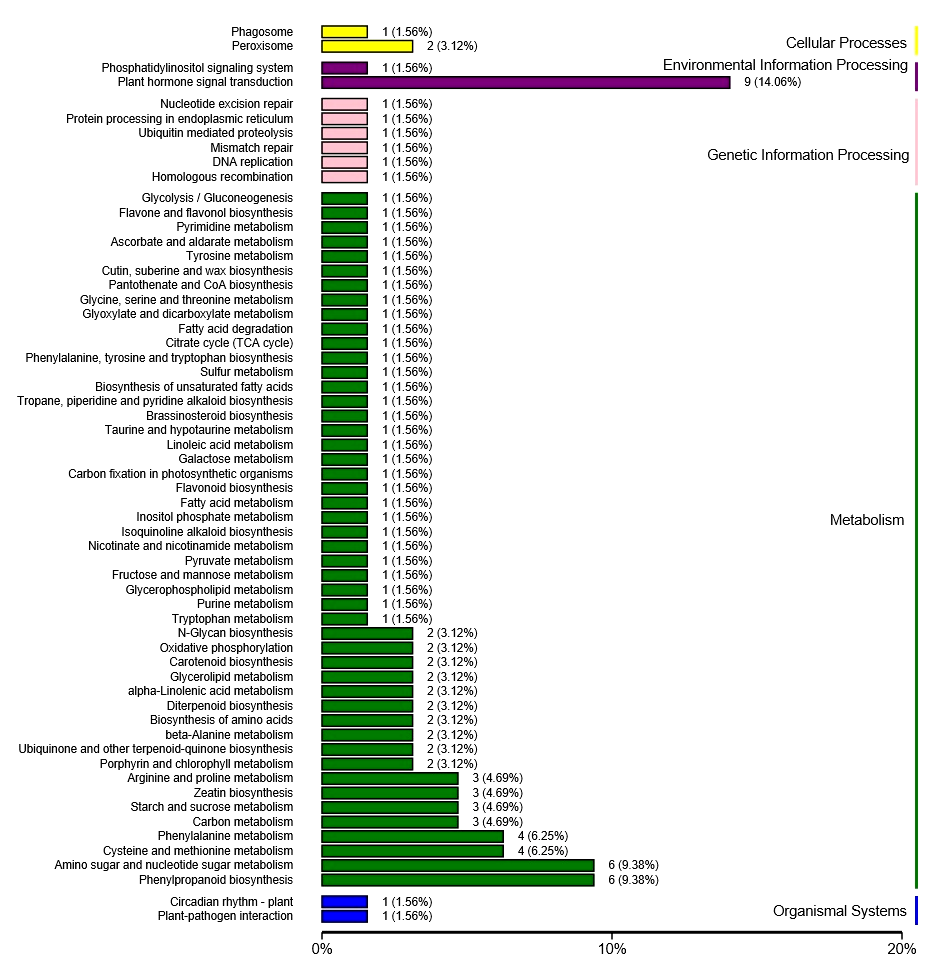


Fig. S4B: KEGG classification of DEGs identified in Zhenghan 10. Vertical axis: KEGG pathway. Horizontal axis: number of genes and proportion of genes in total annotated genes.


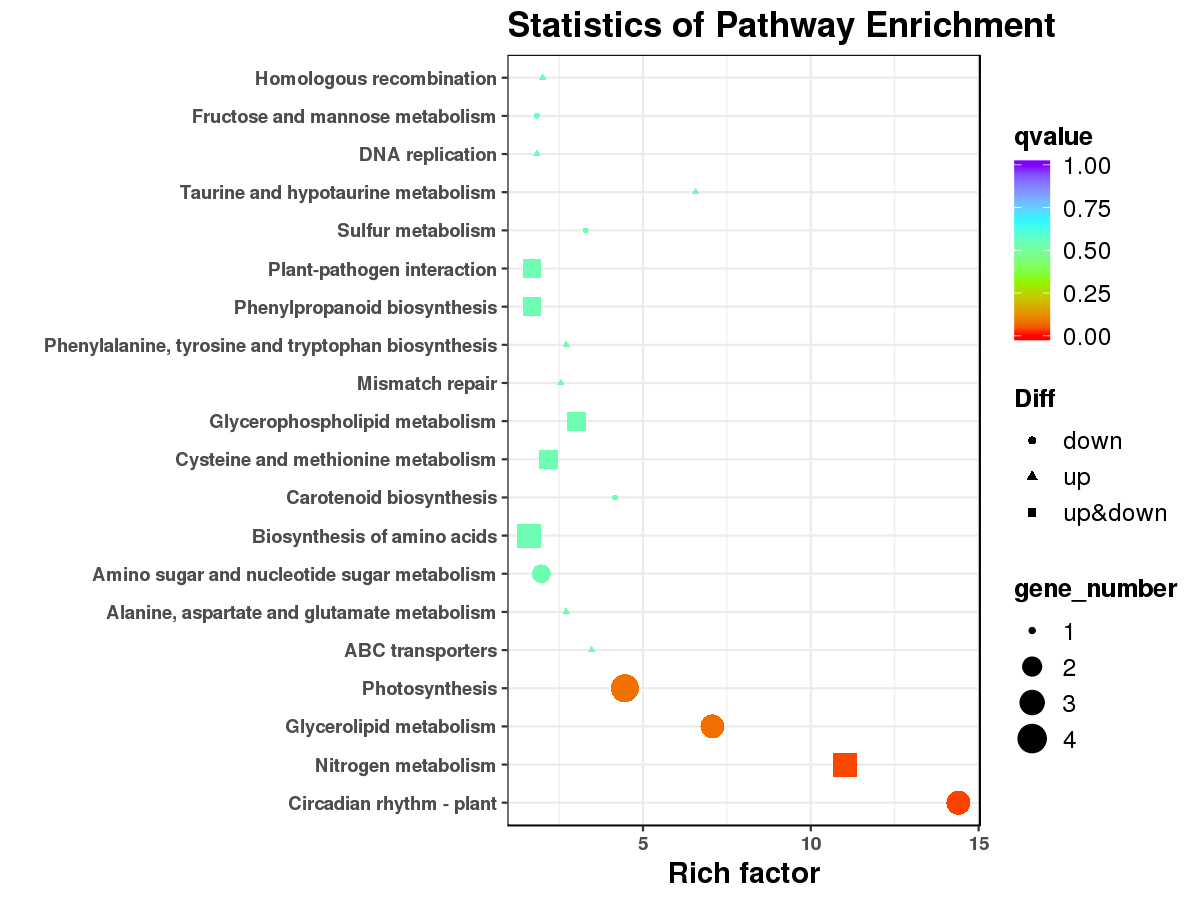


Fig. S5A: KEGG pathway enrichment bubble diagram of DEGs identified in Kasalath. Each circle represents a KEGG pathway, name of which is shown on the left legend. Abscissa is enrichment factors, showing the proportion of x to y, x is the ration of DEGs in a pathway with all DEGs in all pathways, y is the ration of expressed genes in a pathway with all genes in all pathways. The color of circle represents tested q-value. The circle size represents number of DEGs annotated in the pathway.


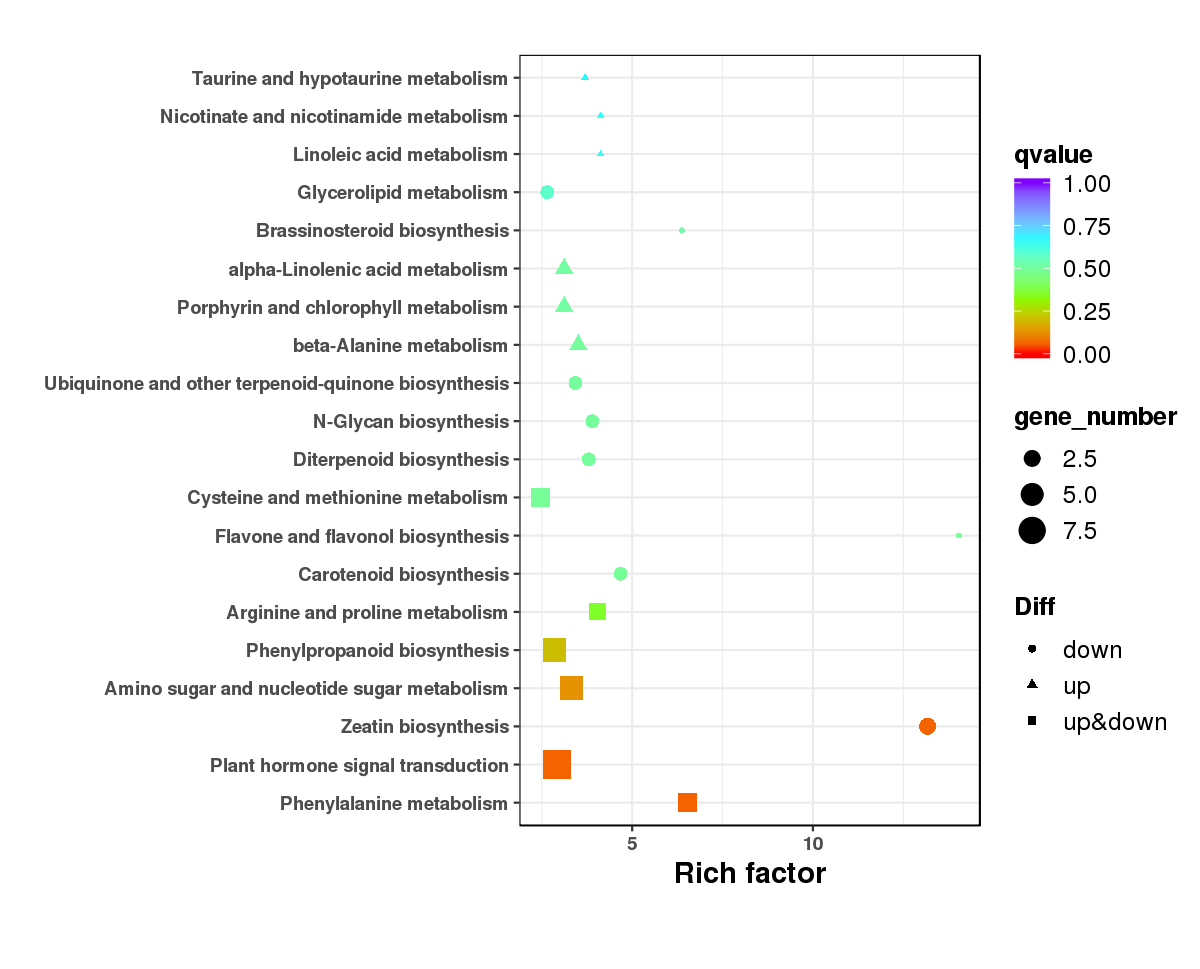


Fig. S5B: KEGG pathway enrichment bubble diagram of DEGs identified in Zhenghan 10. Each circle represents a KEGG pathway, name of which is shown on the left legend. Abscissa is enrichment factors, showing the proportion of x to y, x is the ration of DEGs in a pathway with all DEGs in all pathways, y is the ration of expressed genes in a pathway with all genes in all pathways. The color of circle represents tested q-value. The circle size represents number of DEGs annotated in the pathway.
